# Thermal characteristics of rheumatoid feet in remission: Baseline data

**DOI:** 10.1371/journal.pone.0243078

**Published:** 2020-12-02

**Authors:** Alfred Gatt, Cecilia Mercieca, Andrew Borg, Andrea Grech, Liberato Camilleri, Corene Gatt, Nachiappan Chockalingam, Cynthia Formosa

**Affiliations:** 1 Faculty of Health Sciences, University of Malta, Msida, Malta; 2 Department of Health, University of Malta, Msida, Malta; 3 Faculty of Medicine and Surgery, University of Malta, Msida, Malta; 4 Department of Statistics and Operations Research, Faculty of Science University of Malta, Msida, Malta; 5 Centre for Biomechanics and Rehabilitation Technologies, Staffordshire University, Stoke-on-Trent, United Kingdom; Universita Campus Bio-Medico di Roma, ITALY

## Abstract

**Objectives:**

Studies have shown conflicting characteristic thermographic patterns of the feet in patients with active rheumatoid arthritis (RA). However, to date no studies have compared thermographic patterns of patients with RA in remission and healthy controls. Thus this study aimed to investigate whether the thermal characteristics of the feet of RA patients, in clinical and radiological remission differ to those of healthy controls.

**Methods:**

Using convenience sampling, RA patients were recruited upon confirmed absence of synovitis by clinical examination and musculoskeletal ultrasound. Thermal images of the feet were taken. Each foot was subdivided into medial, central, lateral, forefoot and heel regions. Subsequently, temperatures in the different regions were analyzed and compared to a cohort of healthy adults.

**Results:**

Data from 32 RA patients were compared to that of 51 healthy controls. The Independent samples T-Test demonstrated a significant difference in temperatures in all the regions of the forefoot between RA participants versus healthy subjects (Table 1). Using the One-Way ANOVA test, no significant difference was found between all the forefoot regions (p = 0.189) of RA patients. Independent sample T-test found significant differences in all heel regions between the two groups (Table 2). One-Way ANOVA demonstrated no significant differences (p = 0.983) between the different foot regions (n = 192) of RA patients.

**Conclusion:**

These findings suggest that RA patients in clinical and radiological remission exhibit significantly different feet thermographic patterns compared to healthy controls. This data will provide the basis for future studies to assess whether thermographic patterns change with disease activity.

## Introduction

Rheumatoid arthritis (RA) is characterized by persistent symmetrical inflammation of the small joints of the hands and feet. Early diagnosis and timely treatment of RA can stop disease progression resulting in better outcomes [1]. Despite the availability of various imaging modalities such as musculoskeletal ultrasound (US) and Magnetic Resonance Imaging (MRI), early diagnosis remains a challenge. Additionally, both US and MRI have limitations such as cost, time, acceptability to patients and availability of expertise and equipment.

Medical infrared thermography is a developing technology that is being used in a number of clinical and research scenarios, including the detection of diabetic foot disease [2] and breast cancer [3], amongst others. This technology has the potential to detect minimal skin temperature changes that accompany joint inflammation since modern cameras are very sensitive and accurate. The hand and foot joints are very accessible to thermography. The advantages of this technology are that it is safe, non-invasive and low cost and allows for the rapid and non-invasive recording of radiating energy that is released from the body [4–6]. Indeed, thermography has been applied previously in a number of studies in an attempt to detect ‘hot spots’ that are claimed to be characteristic of this condition. It is claimed that cutaneous temperatures may vary due to inflammation, with these subtle temperature changes being detected by infrared thermography [7]. However, there is conflicting evidence surrounding the ability of thermography to detect joint inflammation, often attributed to the methodologies applied.

In a small study, 8 RA patients exhibited significantly higher hand temperatures ranging from 24.8°C to 36.5°C, compared to the healthy group. Unfortunately, disease activity status was not mentioned in this paper [8]. Likewise Frize et al. [1] conducted a study in 13 RA patients and concluded that it is possible to detect RA using IR imaging. Gizińska an co-authors [9] found significant temperature differences between RA patients and healthy controls and no significant differences between feet. In contrast, Jones et al (2018) [10], performed a cross-sectional study of the hand joints of 49 RA patients and found no statistically significant relationship between joint temperature and clinical assessment of disease activity including the Health Assessment Questionnaire. They concluded that although there may be a role for thermography in the assessment of larger joints, it does not appear to be an effective modality for the small joints of the hand.

Gatt et al [11] report significantly elevated hand and finger temperatures in a cohort of RA subjects without active synovitis compared to healthy adults. Clearly the literature about the effectiveness of thermography in detecting joint inflammation is sparse, especially with regards to foot inflammation. More so, in relation to those RA patients who are in remission. The authors postulate that one cannot apply thermography to detect joint inflammation in RA when the baseline data pertaining to RA itself is unknown. As has been demonstrated [11], patients with RA may exhibit changes in thermographic patterns even in the absence of active inflammation. Hence the need to perform this same research to characterize thermal patterns in the feet of RA patients without active inflammation becomes very clear.

This study aimed to investigate whether the thermal characteristics of the feet of RA patients, in clinical and radiological remission, differ to those of healthy controls. This would make it the first study of its kind, setting a baseline against which future studies in thermography in RA could be compared.

## Methods

Following ethical approval by the University Research Ethics Committee and following informed written consent, 32 participants living with RA were recruited on a first through the door basis from a rheumatology clinic in a general hospital. The Declaration of Helsinki regulations were abided to throughout the study [12]. Additionally, data from a previous study involving 51 healthy adults with no history of significant medical, surgical, vascular or neurological disease, collected utilizing exactly the same protocol for imaging and analysis, was made available for comparison. This study had established baseline thermographic data for healthy adults [13].

Inclusion criteria included adults diagnosed with RA by a consultant rheumatologist according to the 2010 Revised American College of Rheumatology and European League Against Rheumatism (ACR/EULAR) Diagnostic criteria [14]. Patients with significant co-morbidities that could possibly affect thermal emissivity of the feet, such as diabetes mellitus, peripheral arterial disease and neuropathy were excluded from the study, as were patients with a history of heavy smoking. RA patients were examined by two rheumatologists. Hospital records were consulted to exclude the presence of significant clinical co-morbidities. Joint tenderness, swelling and pain, together with a DAS28 score <2.7, were also exclusion criteria. Additionally, a C-Reactive Protein (CRP) <5 was utilized to confirm minimal disease activity. A number of participants also underwent diagnostic ultrasonography by a trained rheumatologist in order to ensure that the recruited participants had no subclinical signs of synovitis. The ultrasonographic test investigated the presence of effusion, synovial hypertrophy and colour doppler [15].

Joint tenderness was assessed by the Ritchie Articular Index (RAI) [16]. If upon the application of the RAI, any joint demonstrated a result >0, it was excluded from the analyses to ensure that all included joints were pain, tender and swelling-free.

**Fig 1:**
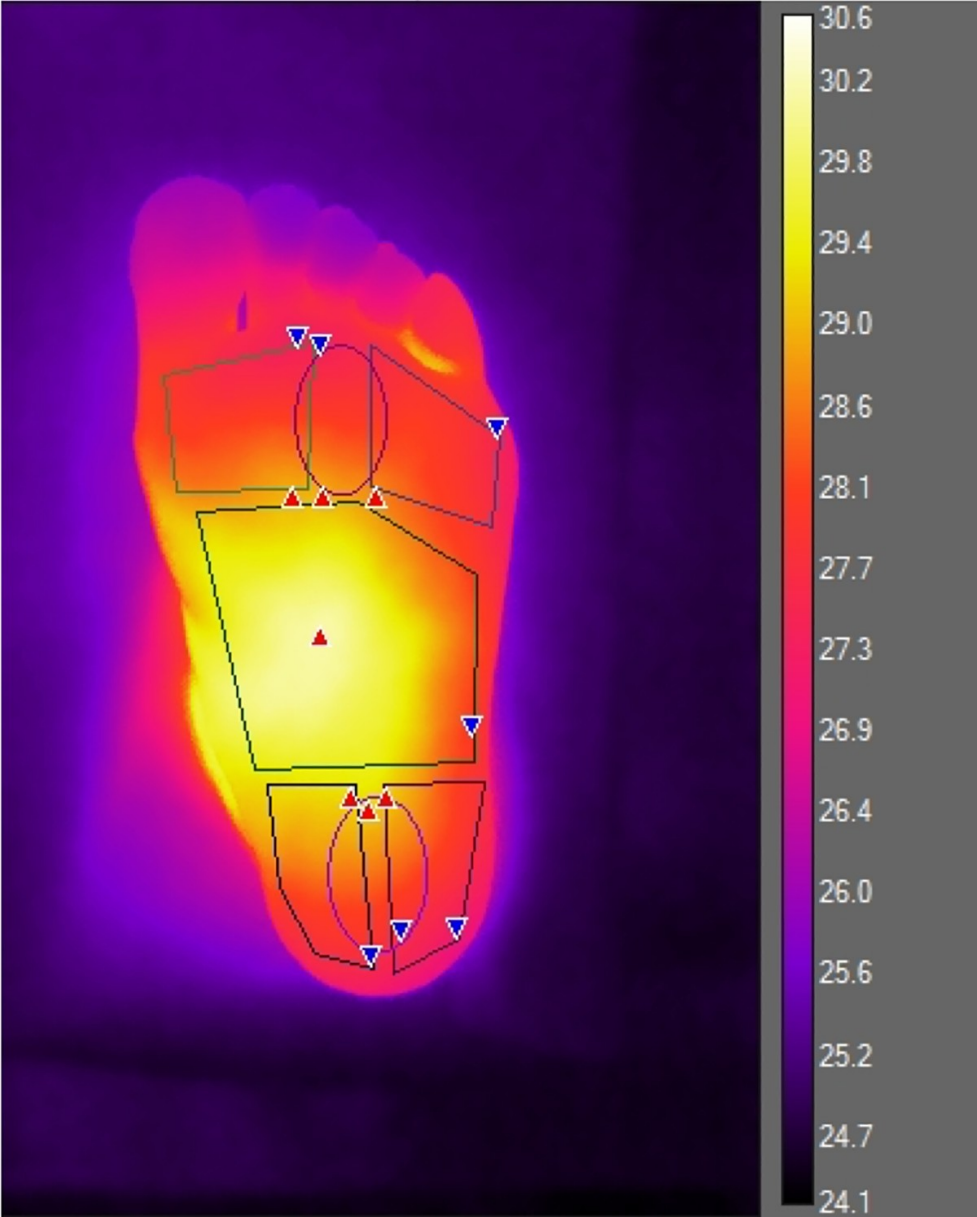
Thermal image of a healthy foot, demonstrating masked regions for analysis.

**Fig 2:**
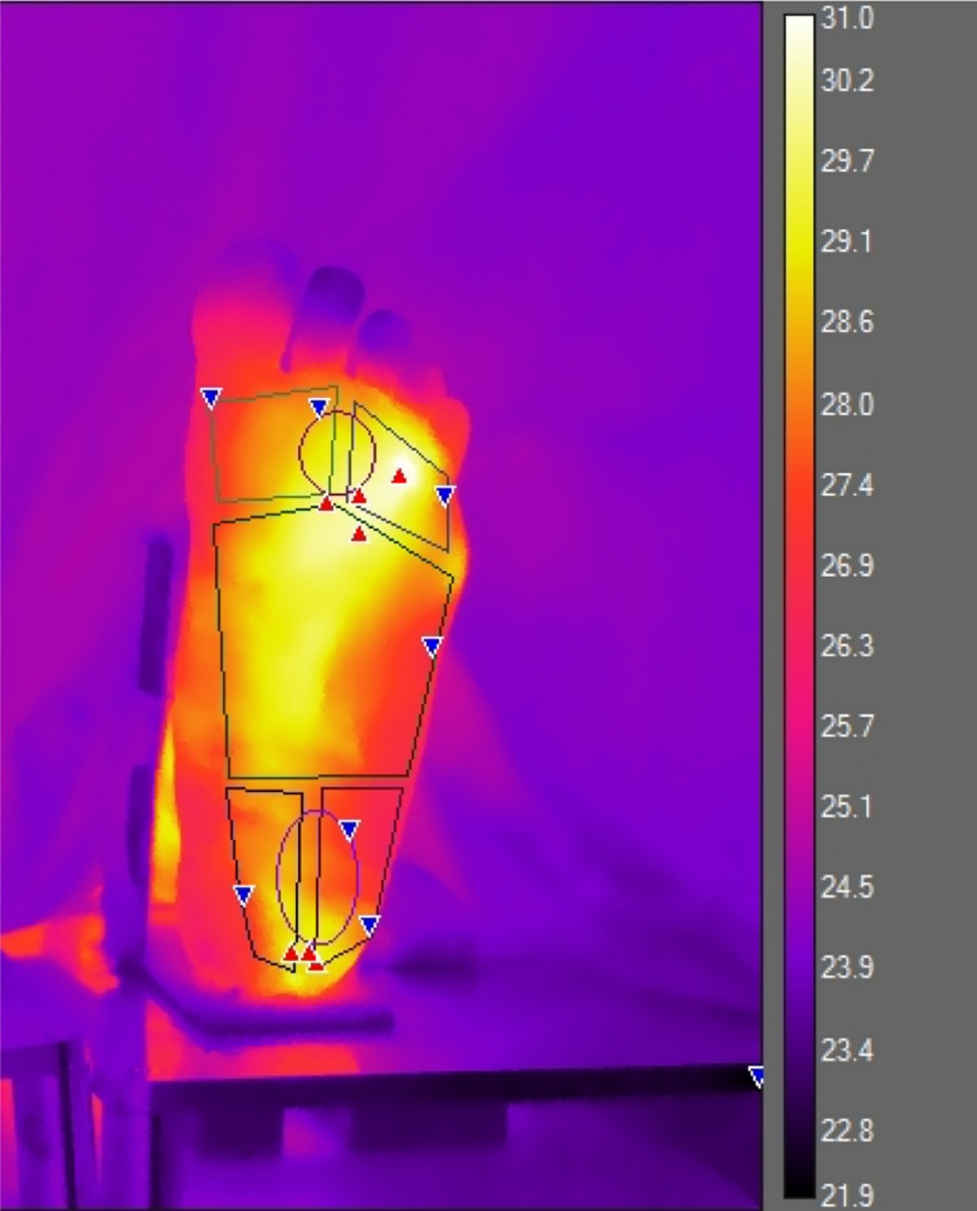
Thermal image of an RA foot, demonstrating masked regions for analysis.

Participants were requested not to apply any topical preparations to their feet on the day of data collection. They were also requested to refrain from eating heavily or smoking significantly before their visit since these could affect the metabolism and therefore skin temperature. Thermographic images were acquired in the same room set at a temperature of 23°C, as per previous literature [13]. After ensuring that any tight clothing was undone, each participant lay supine for a minimum of 15 minutes in order to acclimatize to the room temperature. Images were acquired using a FLIR (Oregon, USA) T630 thermal camera. This is an uncooled microbolometer thermal camera with a Focal Plane Array size of 640x480 and NETD of <40mK at 30°C. The camera was held on a tripod placed 1.5m away and perpendicular to the subject’s feet. Visual and thermal images of the plantar aspect of the feet were taken. These were then analyzed utilizing Flir ResearchIR Max software version 4.30.1.70. Regions of interest from which mean temperatures were extracted included the medial, central and lateral forefeet and heels [13]. Sample images of RA feet and healthy feet are included in Figs 1 and 2 respectively.

Temperature data was analyzed using SPSS v25. The Shapiro Wilk test confirmed the normality assumption of the temperature distributions and parametric tests were used to compare mean temperatures between different regions and different groups of participants. The Independent sample T-Test was used to compare mean temperatures between the two groups and this was carried out for each region separately. The One-Way ANOVA test was utilized to compare mean temperatures between all regions and this was carried out for each group of participants separately. A 0.05 level of significance was adopted for all statistical tests.

## Results

The RA group consisted of 29 females and 3 males, mean age 60.19 years (±12.4); mean height 1.57m (±0.1); mean weight 75.7kg (±21.5). The Healthy group consisted of 51 participants (12 males and 39 females with a mean age of 36years (±12.2), mean weight 70.5kg (±14) and mean height of 1.65m (±0.1).

Mean duration of RA amongst the study group was 15.2years (±11.9). Twenty-nine participants were on conventional Disease Modifying Drugs (DMARDS); out of these, 5 were also on biologics, 4 were also on glucocorticoids, whilst 4 were on a combination of DMARDS + glucocorticoids + biologics. Sixteen patients were on DMARDS alone. One patient was solely on a biologic and another on a biologic and glucocorticoids.

The Independent sample T-Test demonstrated a significant difference in mean temperatures in all the regions of the forefoot between RA participants and Healthy subjects (Table 1), with the RA participants displaying significantly higher mean temperatures than the healthy subjects. The One-Way ANOVA test yielded no significant differences in mean temperatures between the RA forefoot regions (p = 0.189).

**Table 1:** Comparison of forefoot regions between RA and controls (healthy participants).

Region of Interest: n = 102 Healthy regions vs n = 64 RA regions	Mean Temp°C	p-value
Lateral forefoot of RA patients vs controls	28.04 ±1.8	p = 0.004
	26.98 ±2.5	
Central region of forefoot RA patients vs controls	28.59 ±1.8	p = 0.005
	26.84 ±2.4	
Medial region of forefoot RA patients vs controls	28.5 ±1.8	p = 0.005
	26.98 ± 2.5	

The Independent sample t-test found significant differences in mean temperatures in all heel regions between the two groups (Table 2), with the RA participants displaying significantly higher mean temperatures than the healthy subjects. Additionally, the One-Way ANOVA test demonstrated no significant differences (p = 0.983) between all RA Regions (n = 192).

**Table 2:** Independent sample T-Test of heel regions RA vs controls (healthy participants).

Region of interest: n = 102 Healthy regions vs n = 64 RA regions	Mean Temp°C	p-value
lateral heel RA vs controls	27.86 ±1.6	p = 0.001
	26.79 ±2.4	
central heel RA vs controls	27.87 ±1.6	p = 0.002
	26.78 ±2.4	
medial heel RA vs controls	27.91 ±1.6	p<0.001
	26.78 ±2.4	

## Discussion

This is the first study comparing thermographic patterns of the feet of RA patients without inflammation to healthy controls. This is an important exercise, since it sets a baseline against which future studies exploring the effect of joint inflammation may be compared.

Local inflammatory processes affect superficial dermal microcirculation [9] which may be detectable by thermography, making this imaging technique particularly suited as a potential screening tool to detect any changes in skin temperature cause by vascular changes located in the superficial layers of the skin [7].

However, the use of thermography in RA has not been explored sufficiently and the available literature is still conflicting and sometimes contradictory [1, 8–10], resulting in inconclusive results [9]. This has precluded the application of this technology to be utilized in this field, as opposed to other fields in which today this technology is gaining in importance [3, 11]. This may be due to the lack of such “normative data for RA feet” which establish a baseline against which experimental groups in studies may be compared.

Medical thermography research of the feet in this specific population is severely lacking. Our results have clearly shown that RA feet without active synovitis exhibit higher temperatures than healthy individuals. These results concur with Gatt et al [11], who explored joints of the hands and fingers of RA subjects without inflammation and have thus likewise set a baseline for normative RA thermal characteristics of the hands. A recent paper has also found significant differences in the temperature of feet between RA participants with inflammation and healthy participants, however it should be noted that their methodology differed somewhat to this study in that they analyzed temperatures through a dorsal approach [9].

Our findings have two important implications. Firstly, that some underlying sub-clinical disease processes may be occurring in the joints of the feet, undetected clinically by DAS-28, RAI and musculoskeletal ultrasound. The nature of this processes, progression and reversibility still have to be determined through further research, through thermography studies and at a cellular level. Secondly additional research is required to determine whether thermographic patterns change and correlate with disease activity and this can be used to diagnose and monitor disease activity in addition to accepted current medical practice.

Another important finding in our study population is that all investigated regions in the RA group exhibited the similar mean temperatures, suggesting symmetry between limbs, concurring with Gizińska et al [9]. Thus should a particular region, or foot, when compared to the ipsilateral foot, exhibit higher temperatures, this could imply some manner of disease activity that is occurring in that foot with a higher temperature, thus warranting further investigation if done in the clinical or research scenarios. Such hypothesis is already being implemented in the diabetic foot, where a temperature gradient >2°C between feet is suggestive of some form of diabetic foot disease and its identification may predict the risk of ulceration and self-monitoring may reduce the risk of ulceration [17]. Similarly, thermography could potentially be a tool to help detect early disease activity in RA. Medical thermography offers several advantages compared to other modalities including being non-invasive, non-touch (precluding use of disinfecting agents yet limiting cross-infections), reliable, quick and very simple to use, even by the patients themselves as a self-monitoring tool. This becomes a reality with the emerging rapid technology advancements. Nowadays simple thermal cameras can be either added to a mobile phones or inbuilt thermal cameras could be made available. Indeed, this area is an area of study which is being further investigated by the authors.

An important implication regards past studies that investigated the presence of inflammatory processes and compared RA hands and/or feet to healthy individuals, which may have erroneously attributed this higher temperature to inflammation. This is clearly not the case, since our subject population, clearly screened for absence of such inflammation, resulted with the higher temperature characteristics. Thus, in such studies, the authors advocate the use of a control group of actual RA subjects known to be without inflammation, when comparing to any experimental group suspected of having inflammation of any of the joints of their hands and feet.

A limitation of this study is the small sample size and the different age group of the RA population compared to the healthy controls. Repeating this study with a larger sample size, possibly on a multi-centre level utilizing thermal cameras with different technical characteristics, could address this limitation.

The results of this study demonstrate differences in cutaneous temperatures overlying the joints between RA and healthy participants. However, the main reason for this research was to set a baseline for future studies. There are some studies at present regarding the use of thermography to detect joint inflammation, which however have produced inconsistent and sometimes contradictory results. There is clearly the need to perform large, multi-centre randomized clinical trials in order to determine the sensitivity and specificity of this imaging modality to finally establish whether it is actually valid and reliable sufficiently to be applied to clinical practice as a screening tool for the timely detection of joint inflammation.

## Conclusion

This study provides evidence that the feet of patients with RA in remission show higher mean temperatures and characteristic thermographic patterns compared to healthy controls. Additionally, no difference in mean temperatures was noted amongst all the regions in the RA feet, indicating symmetry between the feet. Thermal imaging may have the potential to be used as a clinical and self-monitoring means in order to detect asymmetric changes in temperatures in feet of patients with RA.

## Supporting information

S1 Data (XLSX)
